# Aurora B SUMOylation Is Restricted to Centromeres in Early Mitosis and Requires RANBP2

**DOI:** 10.3390/cells12030372

**Published:** 2023-01-19

**Authors:** Erica Di Cesare, Sara Moroni, Jessica Bartoli, Michela Damizia, Maria Giubettini, Carolin Koerner, Veronica Krenn, Andrea Musacchio, Patrizia Lavia

**Affiliations:** 1 Institute of Molecular Biology and Pathology (IBPM), CNR National Research Council of Italy, 00185 Rome, Italy; 2 Department of Biology and Biotechnology “Charles Darwin”, Sapienza University of Rome, 00185 Rome, Italy; 3 CrestOptics, 00165 Rome, Italy; 4 Max Planck Institute of Molecular Physiology, 44227 Dortmund, Germany

**Keywords:** SUMOylation, Aurora B, in situ proximity ligation assay (isPLA), RANBP2, mitosis

## Abstract

Conjugation with the small ubiquitin-like modifier (SUMO) modulates protein interactions and localisation. The kinase Aurora B, a key regulator of mitosis, was previously identified as a SUMOylation target in vitro and in assays with overexpressed components. However, where and when this modification genuinely occurs in human cells was not ascertained. Here, we have developed intramolecular Proximity Ligation Assays (PLA) to visualise SUMO-conjugated Aurora B in human cells in situ. We visualised Aurora B-SUMO products at centromeres in prometaphase and metaphase, which declined from anaphase onwards and became virtually undetectable at cytokinesis. In the mitotic window in which Aurora B/SUMO products are abundant, Aurora B co-localised and interacted with NUP358/RANBP2, a nucleoporin with SUMO ligase and SUMO-stabilising activity. Indeed, in addition to the requirement for the previously identified PIAS3 SUMO ligase, we found that NUP358/RANBP2 is also implicated in Aurora B-SUMO PLA product formation and centromere localisation. In summary, SUMOylation marks a distinctive window of Aurora B functions at centromeres in prometaphase and metaphase while being dispensable for functions exerted in cytokinesis, and RANBP2 contributes to this control, adding a novel layer to modulation of Aurora B functions during mitosis.

## 1. Introduction

Protein conjugation with SUMO (small ubiquitin-like modifier) peptides, or SUMOylation, reversibly alters the surface of target proteins and, hence, modulates their interactions with partners, localisation, activity and stability, as reviewed by [1,2,3]. SUMO pathway components influence a variety of dynamic processes, e.g., chromatin organisation and remodelling, DNA repair and recombination, RNA transcription and splicing and nuclear/cytoplasmic transport, as reviewed by [4,5,6,7,8]. 

Four SUMO isoforms exist in higher eukaryotes, of which SUMO2 and 3 are 96% identical (henceforth referred to as SUMO2/3). They are produced as precursor proteins that are processed to mature form by specific peptidases, then conjugated to their substrates by specific ligases in a cascade formally resembling that operating in ubiquitination. Multiple SUMO E3 ligases contribute to the targeting specificity of SUMOylated substrates [2,3]. SUMO peptidases, collectively indicated as SENPs, deconjugate SUMO from target proteins and ensure the reversibility of the conjugated state. 

Protein modification by SUMO conjugation/deconjugation is assuming growing importance during mitosis. SUMO pathway components, including E2 and E3 SUMO ligases, as well as SENPs, localise at chromosomes, centromeres and kinetochores during mitosis [9,10,11,12], suggesting that cycles of SUMO conjugation and deconjugation take place at these structures. Indeed, various transiently SUMOylated proteins have been identified and therein reviewed in [13,14,15]. 

RANBP2/NUP358, the largest component of the nuclear pore complex (NPC), harbours a SUMO E3 ligase domain overlapping with a SUMO-interacting motif (SIM) [16]: RANBP2 can therefore conjugate proteins with SUMO via its E3 activity and, via its SIM domain, it can interact with and stabilise proteins SUMOylated by other ligases, conferring an additional layer of control to the SUMOylated state of target proteins. A well-characterised example is RANGAP1, a regulator of the GTPase RAN and a target of SUMOylation [17,18,19] by the SUMO E2 ligase UBC9 [20,21]. RANBP2 interacts with both RANGAP1 and UBC9 via the SIM domain, forming a multimeric complex called RRSU (for RANBP2/RANGAP1-SUMO1/UBC9), that has increased SUMO E3 ligase activity compared to that of RANBP2 alone [22,23,24]. In interphase, the RRSU complex associates with NPCs, where RANBP2 is a resident NUP and regulates the SUMOylated state of certain nuclear transport cargoes [25]. After nuclear envelope (NE) breakdown and NPC disassembly, RRSU localises to the spindle microtubules. A fraction is recruited to kinetochores as microtubules attach to them [26,27,28], where SUMOylated RANGAP1 modulates RAN activity in control of stability of microtubule-kinetochore interactions [26]. RANBP2 inhibition or mislocalisation yield disorganised spindles and chromosome missegregation, promoting genetic instability in cultured cells [26,27,29,30,31] and in animal models [32]. 

SUMOylation also regulates TOP2A recruitment to mitotic centromeres [33], which enables TOP2A decatenation of the DNA in sister centromeres at the chromosome segregation onset [34]. TOP2A conjugation with SUMO requires the E3 ligase PIASγ, which is abundant in chromosomes and is thought to facilitate TOP2A dissociation for chromosome arms [35,36]. RANBP2 is also required, as mutant mice expressing low RANBP2 levels show TOP2A defective SUMOylation, failed localisation at centromeres and impaired sister centromeres decatenation [32]. 

Borealin, a component of the chromosomal passenger complex (CPC), also undergoes SUMO conjugation and deconjugation cycles under the control of RANBP2 as the responsible ligase, while the SUMO isopeptidase SENP3 catalyses deSUMOylation, though the functional significance of these modifications is not clear [37]. Thus, centromeres and kinetochores are active platforms for protein modification with SUMO and RANBP2 is an important player in their SUMOylated state.

The Aurora B kinase, the catalytic component of the CPC, is also a SUMO2/3 conjugation substrate, both in vitro and in transfected human cells overexpressing SUMO pathway components [38,39], as well as in *C. elegans* [40]. Aurora B acts in several mitotic steps: at mitotic onset, it phosphorylates chromosomal proteins and regulates chromosome condensation; it then dissociates from chromosomes and concentrates at centromeres; therein, it phosphorylates centromere- and kinetochore-associated factors to correct microtubule misattachments and promote chromosome biorentation; finally, it relocates from kinetochores to the central spindle and eventually to the midbody at cytokinesis to prepare for cell division. These transitions are critically regulated by sequential interactions with partner proteins in time and space, as reviewed in [41,42,43]. In particular, a series of concerted events orchestrate Aurora B mobilisation from chromosomes and accumulation at centromeres: (i) the CPC localising component INCENP interacts with HP1 (heterochromatin protein 1); (ii) the phosphorylation of histone H3 at Thr3 by the kinase Haspin generates a docking site for the CPC component Survivin; and (iii) the concomitant phosphorylation of histone H2A at Thr120 by the Bub1 kinase recruits Shugoshin (Sgo1), which in turn interacts with and recruits Borealin. Aurora B phosphorylates histone H3 at serine 10 (H3^phSer10^) and serine 28 (H3^phSer28^), which triggers the dissociation of HP1 from phosphorylated chromatin, thus facilitating CPC dissociation from chromosome arms and enrichment at centromeres. These events cooperate to anchor the bulk of the CPC components at centromeres and inner kinetochores. Aurora B becomes fully active at kinetochores, where its kinase activity is itself regulated both by upstream kinases and auto-phosphorylation at key residues. 

Previous studies identified a single SUMO acceptor site within Aurora B: lysine K202 in the human kinase [39] and K207 in the murine orthologue [38] within the conserved sequence IHRDIKPEN, containing the SUMO consensus motif ΦKxE (Φ, large hydrophobic residue; K, acceptor lysine; x, any amino acid; E, glutamic acid). Where and when the Aurora B conjugation with SUMO takes place in human cells still remains unclear. Here, we have developed the intramolecular as the Proximity Ligation Assay (PLA) method to visualise SUMOylated Aurora B during mitotic progression in human cells. We find that Aurora B/SUMO2-3 PLA products accumulate at centromeres in prometaphase and metaphase, then decline in later stages, indicating that SUMOylation is distinctive of Aurora B functions at centromeres in early mitosis but not at cytokinesis. Previous experiments indicated that SUMO ligase PIAS3 was required for Aurora B-SUMO2/3 conjugation [39]. Here we show that RANBP2 also interacts with Aurora B and contributes to Aurora B SUMOylation and concentration at centromeres, suggesting a novel link between RANBP2 and this post-translationally modified form of Aurora B.

## 2. Materials and Methods

### 2.1. Cell Culture, Synchronisation and Treatments

HeLa epithelial cells (ATCC: CCL-2) were grown in a humidified atmosphere at 37 °C in 5% CO_2_ in Dulbecco’s Modified Eagle’s Medium (DMEM) supplemented with 10% foetal bovine serum (FBS), 2% L-Glutamine, 2.5% HEPES and 2% penicillin/streptomycin. Flp-In T-Rex HeLa cells (a kind gift from Prof. Jonathon Pines, the Institute of Cancer Research, London UK) were maintained as described in [44] in DMEM with 10% tetracycline-free FBS supplemented with 50 μg/mL zeocin (ThermoFisher Scientific, Waltham, MA, USA) and 5 μg/mL blasticidin (Sigma-Aldrich, St. Louis, MO, USA). Where indicated, cells were synchronised by culturing for 24 h in 2 mM thymidine to arrest the cell cycle in G1/S transition, then released from arrest in a fresh medium containing 30 μM deoxycytidine to restore the synthesis of deoxyribonucleotide triphosphates. Where indicated, prior to fixation cultures were incubated in S-trityl-L-cysteine (STLC) for 18 h to inhibit kif11, then released for 40 min to recover kif11 activity. Control cultures were incubated with DMSO solvent. 

### 2.2. Plasmids and Mutagenesis

The Aurora B sequence was cloned in the expression vector pcDNA5/FRT/TO. Aurora B constructs were rendered RNAi resistant by inserting neutral mutations [45]. To generate Aurora B^K202R^, site-directed mutagenesis (QuickChange; Agilent Technologies) was performed according to the manufacturer’s instructions. C-terminal EGFP-Aurora B fusions were generated by PCR amplifying the EGFP sequence from pcDNA5/FRT/TO EGFP-IRES vector and cloning in frame with the Aurora B sequence in pcDNA5/FRT/TO by Restriction Free (RF) Cloning (http://www.rf-cloning.org/index.php) [46]. The control EGFP plasmid was generated by PCR amplifying the EGFP sequence from pEGFP-C1 (Takara Bio Europe SAS, Saint-Germain-en-Laye, France) and cloning in pcDNA5/FRT/TO. pcDNA5/FRT/TO contains a flippase (FLP) recombination target (FRT) site, which enabled integration into a homologous site in a HeLa-derived cell line (Flip-in line). Plasmid sequences were verified at Bio-Fab Research s.r.l., Rome, Italy. 

### 2.3. Generation of Stable Inducible Cell Lines

Flp-In T-Rex HeLa cells containing Aurora B-EGFP constructs were generated following the ThermoFisher protocol (Flp-In™ T-REx™ Core Kit, ThermoFisher K650001; ThermoFisher Scientific). Briefly, cells were co-transfected with Aurora B-GFP constructs (pcDNA5/FRT/TO) and pOG44 (expressing Flp recombinase) in 1:9 ratio using Fugene HD (Promega). Forty-eight hours after transfection, Flp-In T-Rex HeLa cells were put under selection for two weeks in DMEM with 10% tetracycline-free FBS supplemented with 250 μg/mL hygromycin (Sigma-Aldrich) and 5 μg/mL blasticidin (Sigma-Aldrich). The resulting foci were pooled and tested for expression. Gene expression was induced with 1 μg/mL doxycycline (Santa Cruz Biotechnology Europe, Heidelberg, Germany).

### 2.4. RNA Interference

Small interfering RNAs (siRNAs) were: RANBP2, 5′-GGACAGUGGGAUUGUAGUGTT-3′; Aurora B, 5′-CGCGGCACUUCACAAUUGATT-3′; and luciferase (GL2), 5′-CGUACGCGGAAUACUUCGA TT-3′ (all from Ambion, Inc., ThermoFisher Scientific). Borealin-specific siRNAs, sc-60277, were from Santa Cruz Biotechnology, Inc. RNA interference conditions were as reported: RANBP2 [27]; Aurora B [47,48]; and Borealin [49]. siRNA concentrations were: RANBP2, 150 nM; Aurora B, 50 nM; and Borealin, 100 nM. siRNAs diluted in OptiMem were transfected using Oligofectamine (Invitrogen).

### 2.5. Immunofluorescence (IF)

Cells grown on polylysine-coated coverslips were fixed in 3.7% paraformaldehyde (PFA)/30 mM sucrose, permeabilised with 0.1% Triton X- 100, then processed for IF using primary and secondary antibodies listed in Supplementary Table S1. Blocking and antibody incubation were at room T° in PBS/0.05% Tween 20/3% bovine serum albumin. DNA was stained with 0.1 μg/mL 4,6-diamidino-2-phenylindole (DAPI, Sigma). Coverslips were mounted in Vectashield (Vector Laboratories, Burlingame, CA, USA).

### 2.6. Proximity Ligation Assay (PLA)

PLA were performed according to the Olink Bioscience’s protocol using Duolink kit reagents. After blocking and incubation with primary antibodies (Supplementary Table S1), the PLA probes anti-mouse MINUS and anti-rabbit PLUS were diluted 1:5 in 1× PBS/0.05% Tween 20/3% BSA and incubated (1 h, 37 °C) in a pre-heated humidity chamber. Hybridisation, ligation and detection were performed as described [50,51], using two alternative Duolink Detection kits (Sigma-Aldrich): the DUO92007 kit, containing a fluorescent detection probe (λ excitation 554 nm, λ emission 576 nm) visualised under the same filter as Cy3 (orange), and the DUO92008 kit, containing a detection probe with λ excitation 594 nm and λ emission 624 nm, visualised using the same filter as Texas Red (red).

### 2.7. Microscopy

Fixed samples were analysed under a Nikon Eclipse 90i microscope equipped with a Qicam Fast 1394 CCD camera (Qimaging). A 100× immersion oil objective (NA 1.3) was used for single-cell images and a 40× objective (NA 0.75) to acquire entire fields. Image acquisition was performed using NIS-Elements AR 4.0 and 4.2 (Nikon); 3D deconvolution of 0.3–0.4 μm z-serial optical sections was performed using the AutoQuant deconvolution module of NIS-Element AR 4.0/4.2. Creation of image projections from z-stacks was performed using the Maximum Intensity Projection (MIP, for quantitative analyses) and Extended Depth of Focus (EDF) functions of NIS-Element AR 4.0/4.2. PLA signal dots were counted using “spot detection” and “count objects” tools of NIS-Element AR 4.0/4.2 [50]. Quantitative analysis of IF signals was measured using NIS-Element AR 4.0/4.2 (nd2 file format), the external background correction was applied and the sum intensity of signals was measured and analysed using Instat3 software.

### 2.8. Western Immunoblotting 

Cells were lysed in RIPA buffer (50 mM Tris-HCl pH 8, 150 mM NaCl, 1% NP40, 1 mM EGTA, 1 mM EDTA, 0.1% SDS, 0.25% sodium deoxycholate) with protease (05892791001 Roche, Basel, Switzerland) and phosphatase (PhoSTOP, 04906837001 Roche) inhibitors. Forty μg of protein extract per lane were loaded onto SDS-PAGE and transferred to nitrocellulose filters (Protran BA83, Whatman, GE Helthcare, Chicago, IL, USA) using a semi-dry system (BIO-RAD). Blocking and antibody incubation were in TBS (10 mM Tris-HCl pH 7.4, 150 mM NaCl) with 0.1%Tween 20 and 5% low fat milk. Primary and secondary antibodies were incubated for 1 h at room T and revealed using ECL (GE Healthcare) on Hyperfilm-ECL films (GE Helthcare).

### 2.9. Statistical Analysis

All experiments were repeated three times unless indicated otherwise and mean values and standard deviations (SD) were calculated. Data were analysed using GraphPad Prism 8. The following tests were employed: multiple χ^2^ test with contingency tables to compare the frequency of categorical variables, usually represented in this study with histograms, and a Mann–Whitney non-parametric test to compare continuous values, which in most experiments had a non-normal distribution across samples. Values were measured in single cells and their distribution in analysed samples is represented in the box plots.

## 3. Results 

### 3.1. Aurora B-SUMO2/3 Ligation Products Can Be Visualised by PLA at Centromeres/Kinetochores in Intact Human Cells 

**Figure 1:**
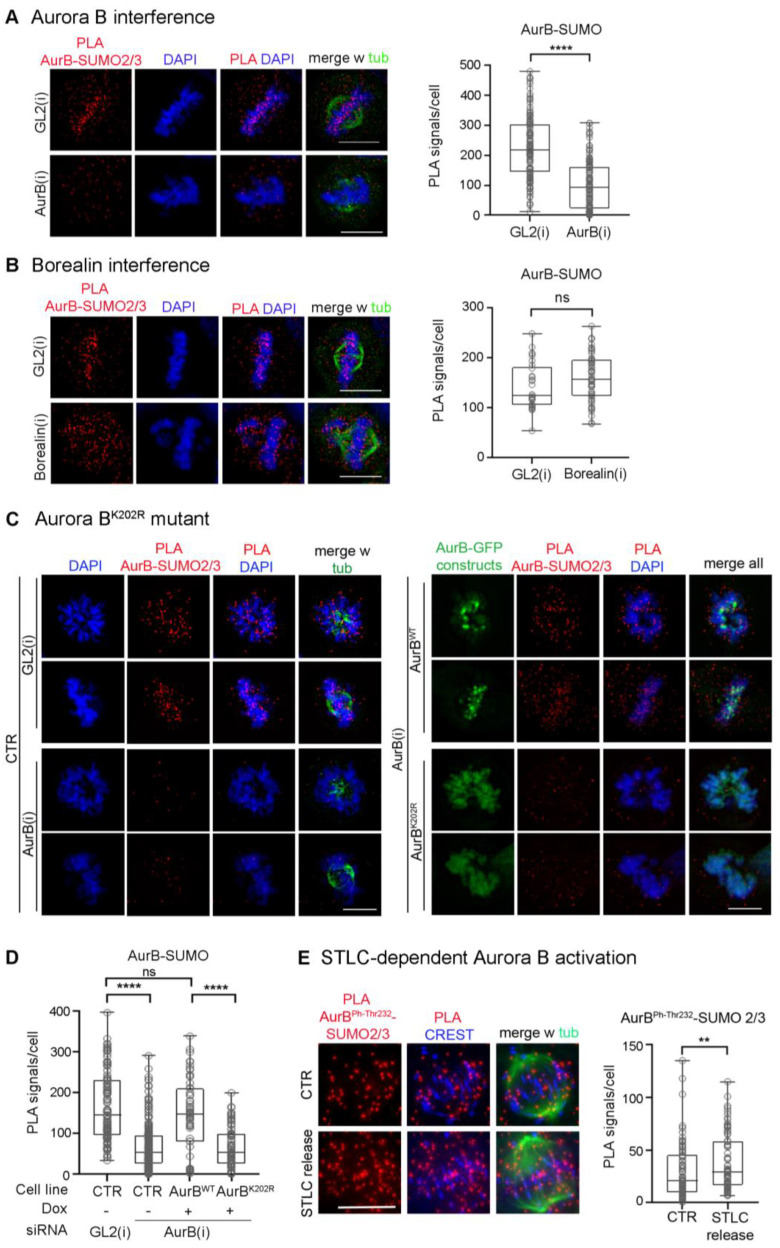
Aurora B-SUMO2/3 ligation products in prometaphase and metaphase HeLa cells. (**A**) Aurora B-SUMO2/3 PLA signals disappear in cells interfered for Aurora B, indicated as AurB(i). Control cultures were treated for RNA interference with neutral siRNAs targeting the firefly luciferase, indicated as GL2(i). The box plot represents the distribution of PLA signals in prometaphase and metaphase in control vs. Aurora B-interfered cultures (140 counted cells/group in 3 experiments); ****, extremely highly significant difference (*p* < 0.0001, Mann–Whitney test). (**B**) Aurora B-SUMO2/3 PLA signals persist in Borealin-interfered cells, which display extensive chromosome misalignment. The box plot on the right shows the abundance of Aurora B-SUMO 2/3 PLA signals/cell as described in (**A**) (60 counted cells/group, 2 experiments). ns, non-significant differences. (**C**) Mutation of K202 abolishes Aurora B PLA signals with SUMO2/3 peptides. Left: Aurora B-SUMO2/3 PLA products in control (CTR) HeLa cultures interfered with neutral (GL2) or with Aurora B-specific siRNAs: the latter abolished the PLA signals. Right: after interference to endogenous Aurora B, PLA assays depict SUMO2/3 products with dox-inducible Aurora B only in the cell line expressing wild-type kinase, but not Aurora B^K202R^ mutant. (**D**) Quantification of Aurora B and SUMO2/3 PLA signals in the cell lines indicated in (**C**). + and − indicate dox addition. At least 60 cells per sample were counted (2 experiments). ns, non-significant difference; ****, *p* < 0.0001. (**E**) Aurora B^phThr232^-SUMO2/3 PLA products under conditions that increase merotelic attachments. The box plot quantifies Aurora B^phThr232^-SUMO2/3 PLA signals in controls (60) and STLC-released (86) cells (2 experiments): PLA signals increase in parallel with the increase in Aurora B^phThr232^ (**, *p* < 0.01). The Mann–Whitney test was used for all comparisons.

In previous work, SUMO-conjugated Aurora B was identified by Western immunoblotting after in vitro reactions with added purified components and in extracts from transfected cells overexpressing SUMO pathway components [38,39], but the occurrence of SUMOylated Aurora B under physiological conditions has not been detected. To assess whether endogenous Aurora B can be visualised in the SUMO-conjugated form in intact cells, here we optimised intramolecular Proximity Ligation Assays (PLA) protocols, previously adapted to visualise SUMOylated topoisomerase II [28], using primary antibodies against Aurora B and SUMO2/3 peptides. PLA signals were detected in mitotic cells at the level of CREST-stained kinetochores (Figure 1A): Aurora B depletion by RNA interference (RNAi) abolished the signals visualised in controls incubated with neutral small interfering RNAs (siRNAs) targeting the firefly luciferase gene, henceforth indicated as GL2. Positive control tests for the specificity of PLA reactions depicted signals using Aurora B and each of its partners, Borealin and INCENP (Figure S1A). In contrast, no PLA signal was generated using gamma-tubulin, which interacts with Aurora A but not Aurora B (Figure S1B).

PLA, in addition to being truly interacting partners, can detect closely neighbouring proteins. Since Borealin is also conjugated with SUMO2/3 in mitosis [37], it was important to verify that our intermolecular PLA protocol truly detects genuine SUMO2/3-conjugated Aurora B, rather than products formed by Aurora B and SUMO2/3 peptides conjugated to the neighbouring Borealin. We designed three independent types of controls to rule out that possibility. First, we repeated the PLA assays in Borealin-depleted cells after RNA interference. Borealin silencing induced severe chromosome misalignment, as expected (Figure S1C); nevertheless, Aurora B-SUMO2/3 PLA signals remained abundant under these conditions (Figure 1B), indicating they were not generated by SUMO groups conjugated to Borealin. 

Second, we repeated the PLA assays using an Aurora B mutant in the conserved lysine K202, which was previously identified as the SUMO target site in assays with overexpressed SUMO2 peptide and PIAS 3 SUMO ligase [39]. We mutagenised the codon encoding the K202 residue to R in the human Aurora B cDNA sequence and introduced synonymous substitutions to render the sequence resistant to siRNAs against the endogenous Aurora B [45], then used the HeLa Flip-in system to integrate either wild-type or K202R Aurora B sequences in frame with EGFP downstream of a doxycycline (dox)-inducible promoter (map in Figure S2A). By Western blot analysis, both wild-type and mutant constructs were selectively expressed in dox-induced cells silenced for the endogenous Aurora B (Figure S2B). Monitoring the construct expression by videorecording showed that the EGFP signal appeared in both cell lines around 3 h after dox addition (Figure S2C). This, combined with cell cycle synchronisation protocols, enabled us to analyse Aurora B wild-type and K202 mutant in G2 and M cells in which Aurora B is physiologically expressed. PLA assays in Flip-In cell lines silenced for the endogenous kinase depicted no PLA signal between dox-induced Aurora B^K202R^ and SUMO2/3 peptides, similar to Aurora B-silenced cultures (Figure 1C, left panels). Aurora B^WT^ yielded abundant signals, comparable to those seen in GL2-interfered controls (Figure 1C, right panels; quantified in Figure 1D). 

Finally, we repeated the PLA assays under conditions that activate the endogenous Aurora B. To achieve this, HeLa cells were treated with STLC, a specific inhibitor of the kinesin Kif11/EG5 that prevents centrosome separation and arrests cells in prometaphase with monopolar spindles; after STLC wash-out, Kif11 activity is resumed and the process of bipolar spindle formation is accompanied by an increase in merotelic attachments compared to physiological mitosis, a condition that stimulates Aurora B activity to facilitate their removal [52,53]. Under these conditions, we found a moderate yet significant increase in Aurora B^phThr322^, marking Aurora B activity, compared to untreated controls (Figure S2D). In parallel, we observed a moderate yet statistically significant increase in Aurora B^phThr23^2-SUMO2/3 PLA signals (Figure 1E). These experiments indicate that Aurora B phosphorylation and SUMOylation coexist, with both modifications progressing in parallel when Aurora B activity is induced. 

Collectively, these assays indicate that the intramolecular PLA protocol depicts genuinely SUMOylated Aurora B kinase at centromeres in intact cells.

### 3.2. SUMOylated Aurora B Is Restricted to the G2-to-Metaphase Window 

**Figure 2:**
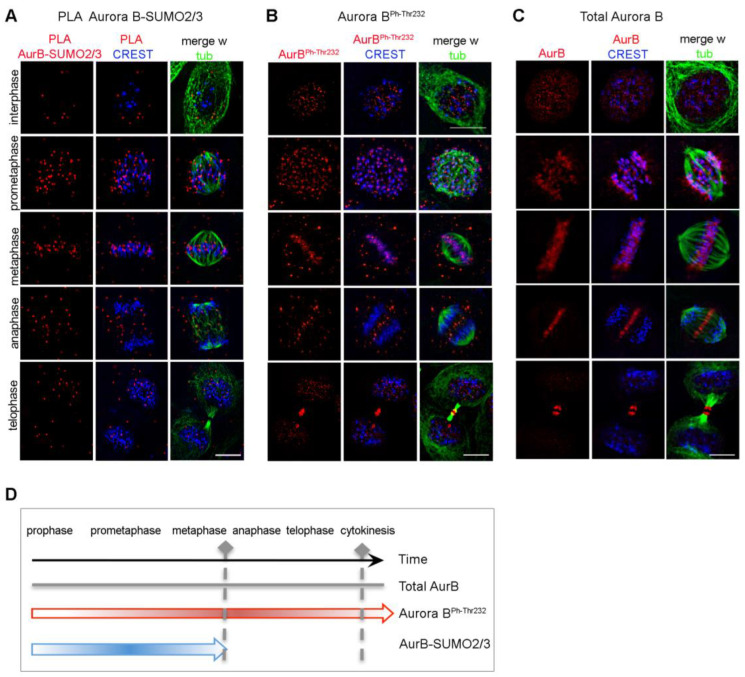
Distribution of Aurora B-SUMO2/3 PLA products in mitotic cells in situ. (**A**) Aurora B-SUMO2/3 PLA signals (red) co-localise with kinetochores (CREST, blue) in prometaphase and metaphase, and decrease abruptly in anaphase/telophase. (**B**) Localisation of active Aurora B, auto-phosphorylated at Thr232. (**C**) Canonical localisation of the total Aurora pool (red) at kinetochores (CREST, blue) and midbody (alpha-tubulin, green) during mitotic progression. Bar, 10 µm. (**D**) The schematics summarises the temporal distribution of Aurora B forms during mitotic progression.

We next determined the timing of Aurora B-SUMO2/3 product appearance in synchronised HeLa cell cultures by thymidine arrest/release to enrich in mitotic stages. PLA signals for Aurora B-SUMO2/3 were observed in G2 cells, increased during prometaphase and accumulated at CREST-stained centromeres during progression to metaphase, gradually aligning between microtubule plus ends and kinetochores (Figure 2A). As cells progressed through anaphase, the abundance of PLA products decreased and those few signals that remained visible tended to assume a non-specific distribution. At cytokinesis, the midbody was virtually devoid of Aurora B-SUMO2/3 products. In parallel assays, signals for the phosphorylated, active Aurora B^phThr232^ persisted throughout mitosis (Figure 2B), accumulating at centromeres and later at the central spindle and midbody, following the distribution of the total Aurora B pool (Figure 2C), as expected. Thus, Aurora B-SUMO2/3 PLA products do not follow the entire span of Aurora B activity during mitosis, but are restricted to early mitotic stages until metaphase (summarised in Figure 2D). In summary, Aurora B-SUMO2/3 products are restricted to the prometaphase-metaphase window, suggesting that Aurora B functions at the midbody in cytokinesis do not require SUMOylation.

### 3.3. Mutation of Lysine K202 Impairs Multiple Aspects of Aurora B Function

**Figure 3:**
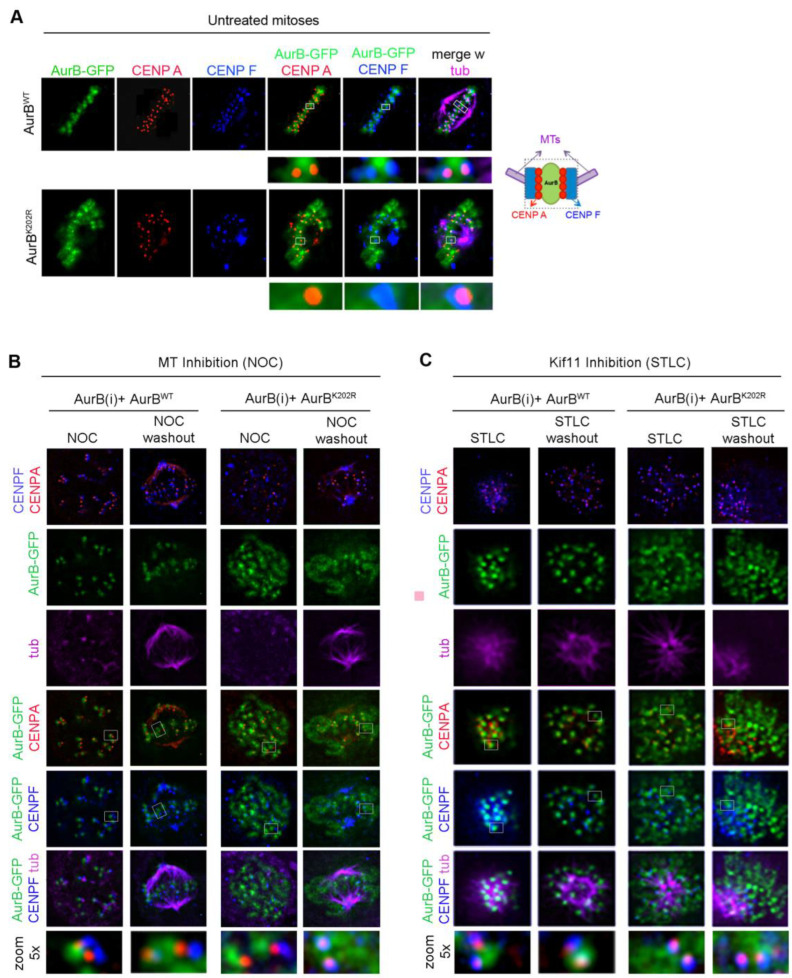
Aurora B^K202R^ does not concentrate at centromeres. (**A**) Aurora B^WT-^GFP (top panel) and Aurora B^K202R^-GFP (bottom panel) in metaphase cells silenced for the endogenous Aurora B. In the blow-up (4×), Aurora B^WT^-GFP has a punctuated appearance bordered by CENP-A and, more externally, CENP-F (see schematics). Aurora B^K202R^-GFP (bottom panel) shows a more diffuse pattern over chromosomes. (**B**) Aurora B^K202R^ fails to concentrate near CENP-A, independent of the absence (NOC) or presence (NOC release) of kinetochore attachments to microtubules. (**C**) Localisation of Aurora B, CENP-A and CENP-F, in kif11-inhibited cells (STLC) with unseparated poles and stretched kinetochores, or in STLC-released cells with stimulated Aurora B activity. Under all conditions, the K202R mutation impairs Aurora B concentration at centromeres, as can be appreciated in the magnification (bottom panels).

In our assays, we noticed that Aurora B^K202R^ was not only impaired in its ability to form ligation products with endogenous SUMO2/3 peptides, but also showed a more diffuse distribution compared to Aurora B^wt^, with much of the EGFP signal remaining spread at chromosomes. To assess this more precisely, we used CENP-A and CENP-F as markers of the inner centromere and outer kinetochore, respectively, in Aurora B^wt^-EGFP and Aurora B^K202R^-EGFP cell lines after silencing the endogenous Aurora B. In metaphase, Aurora B^WT^-EGFP had a sharp, focused localisation at the inner centromere close to CENP-A, while CENP-F remained more external (Figure 3A, top panel). In Aurora B^K202R^-EGFP cells, the EGFP signal was not sharply recruited at centromeres and a large fraction over chromosome arms (Figure 3A, bottom panel). We examined kinetochores devoid of tension in cells treated with nocodazole (NOC) to inhibit microtubule polymerisation: under these conditions, Aurora B^WT^ neighboured CENP-A or overlapped with it due to the lack of stretching of unattached kinetochores; after NOC wash out, punctuated Aurora B^WT^-EGFP signals resumed an aligned configuration juxtaposed to CENP-A (Figure 3B). Aurora B^K202R^-EGFP signals instead showed a persistent association with chromosomes, with no obvious difference in NOC or after wash-out. We also treated the cells with STLC to stimulate Aurora B activity after inhibition and resumption of the kinesin Kif11/EG5 activity (see Figure S2D). Even under these conditions, Aurora B^K202R^-EGFP still remained largely associated with chromosomes (Figure 3C). Thus, Aurora B^K202R^ fails to concentrate at centromeres, independent of kinetochore tension or interactions with microtubules, and is also insensitive to the requirement for increased Aurora B-governed correction activity at kinetochores. 

The impaired localisation of AuroraB^K202R^ at centromeres did not reflect a failure to interact with CPC partners: Aurora B^K202R^ remained proficient for the interaction with INCENP in PLA assays (Figure S3A, compared to Figure S1A). Consistent with this, INCENP and Aurora B^K202R^-EGFP signals were found to co-localise along chromosomes in parallel IF experiments (Figure S3B). Borealin also retained a chromosome-associated fraction in most of Aurora B^K202R^-expressing metaphase cells (Figure S3C). Thus, AuroraB^K202R^ effectively interacts with CPC partners and actually retains a fraction of them at chromosomes. We remarked that both INCENP and Borealin localised normally at the central spindle and midbody in telophase in AuroraB^K202R^ cells, indistinguishable from their localisation in Aurora B^WT^-expressing cells, indicating that SUMOylation is dispensable for localisation of Aurora B and its CPC partners in late mitosis, consistent with the observation that Aurora B SUMOylation is physiologically lost in those phases (Figure 2A). 

**Figure 4:**
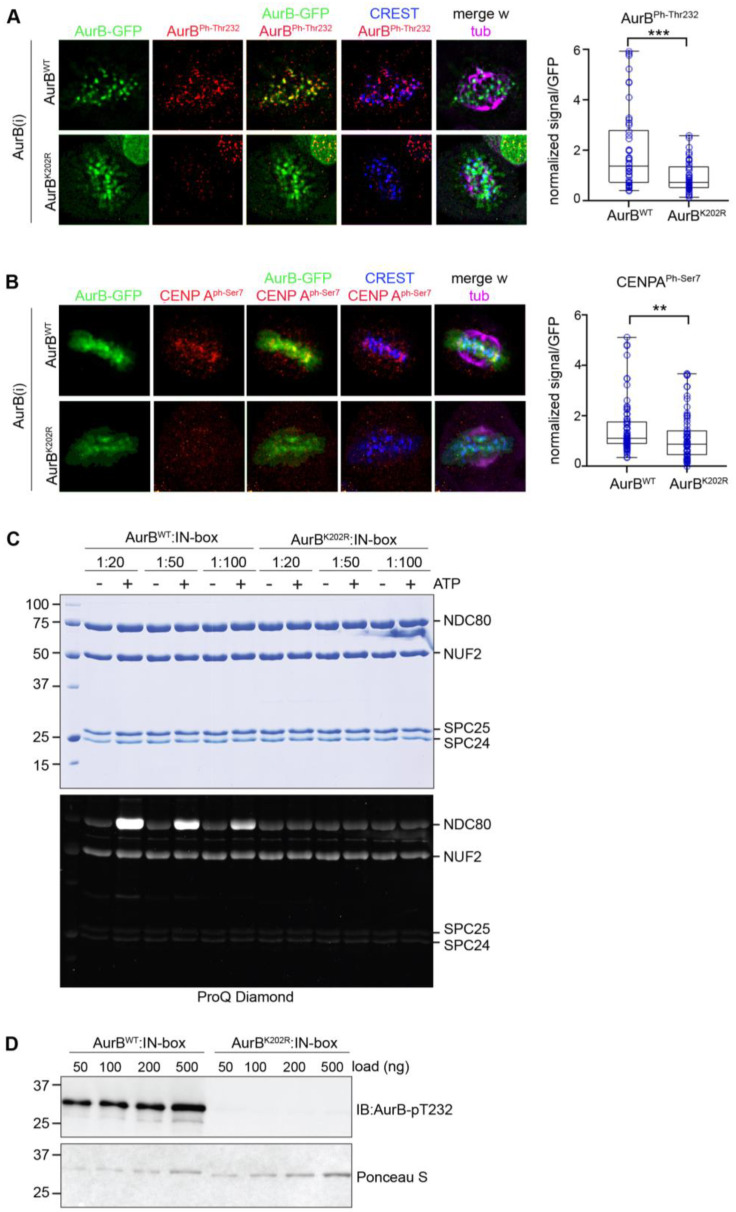
Impaired Aurora B and CENP-A phosphorylation at centromeres in the Aurora B^K202R^-expressing cell line. (**A**) After endogenous Aurora B silencing, Aurora B^Ph-Thr232^ is depicted (red channel) at CREST-stained kinetochores in Aurora B^WT^-expressing metaphases, but substantially decreases in cells expressing Aurora B^K202R^ mutant. Exogenous Aurora B proteins are visualised from the GFP signal. Data from three experiments in the wild-type vs. mutant Aurora B expressing cell lines are quantified in the bow plot (***, *p* < 0.001) (**B**) CENP-A^Ph-Ser7^ (red) is visible at metaphase centromeres in Aurora B^WT^- but decreases in Aurora B^K202R^-expressing cells. Data are quantified in the box plot (three experiments), (**, *p* < 0.01). (**C**) Aurora B^K202R^ is not proficient for phosphorylation of NDC80 in vitro (left panel). After phosphorylation reactions with purified components at the indicated concentrations, ProQ Diamond staining visualises a dose-dependent increase in phosphorylated NDC80 with Aurora B^WT^/INCENP. No phosphorylated bands are seen with Aurora B^K202R^/INCENP (compare to the -INCENP, -ATP lanes). (**D**) Purified Aurora B^WT^, but not Aurora B^K202R^, is reactive to Aurora B^Ph-Thr232^ antibody, indicating self-phosphorylation in vitro.

Studies of the murine protein found that non SUMOylatable Aurora B^K207R^ mutant (homologous to human Aurora B^K202R^) remained catalytically active [38]. In contrast, a study of the human Aurora B showed that SUMOylation reinforced the kinase catalytic activity in vitro and that non SUMOylatable Aurora B^K202R^ was devoid of kinase activity [39]. In our experiments we examined Aurora B^Ph-Thr232^ (Figure 4A), and phosphorylated CENP-A (PhSer7) (Figure 4B) as reporters of Aurora B activity: we found a significant decrease in both markers at kinetochores in Aurora B^K202R^-expressing cells silenced for the endogenous kinase, compared to Aurora B^WT^-expressing cells. This could be attributed in part to the impaired concentration of Aurora B^K202R^ at centromeres, yet the severe failure of Aurora B self-phosphorylation and CENP-A phosphorylation observed in our experiments prompted us to assess the kinase activity of Aurora B^K202R^ in vitro. In Figure 4C Aurora B^wt^ phosphorylates its substrate NDC80 (also known as HEC1) in the presence of the kinase-activating fragment of INCENP (the IN-box), in an ATP- and dose-dependent manner, as expected (left panel). Under the same conditions, Aurora B^K202R^ yielded no detectable HEC1 phosphorylation. Furthermore, probing the Aurora B^K202R^ mutant with anti-Aurora B^Ph-Thr232^ antibody detected no signal (Figure 4D), indicating that the mutant does not autophosphorylate. Together, these experiments show that the K202 site in human Aurora B simultaneously controls Aurora B SUMOylation, concentration at prometaphase/metaphase centromeres and catalytic activity. AuroraB^K202R^ therefore does not support a separation of function analysis and cannot disentangle whether SUMOylation directly regulates Aurora B localisation and kinase activity. 

### 3.4. RANBP2 Contributes to the SUMOylated State of Aurora B

**Figure 5:**
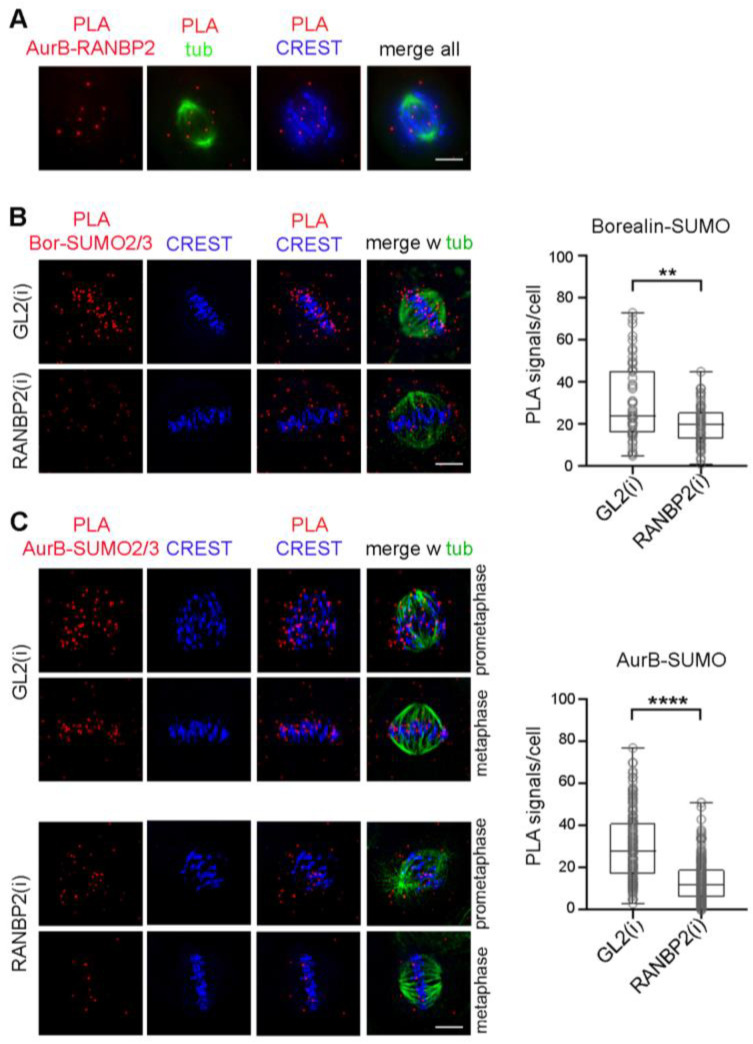
RANBP2 is required for Aurora B-SUMO2/3 PLA products. (**A**) RANBP2 and Aurora B form PLA products in mitotic cells. A representative late prometaphase is shown, in which PLA signals are juxtaposed to kinetochores (CREST, blue). (**B**) Borealin-SUMO2/3 PLA reactions in a control metaphase treated with neutral siRNAs (GL2) and, below, in a RANBP2-interfered cell. The box plot represents Borealin-SUMO2/3 PLA signals per cell in control (n, 55) and RANBP2-interfered (n, 65) metaphases in 2 experiments (**, *p* < 0.01). (**C**) Aurora B-SUMO2/3 PLA signals were analysed under the same conditions as above, in control (GL2) and RANBP2-interfered cells. Exemplifying prometaphases and metaphases are shown. The box plot represents Aurora B-SUMO2/3 PLA signals per cell in controls (n, 150) and RANBP2-interfered (n, 200) cells in 5 experiments; ****, highly significant difference (*p* < 0.0001).

Biochemical assays using synchronised cell extracts showed that RANBP2 is required for SUMO conjugation of Borealin, and that this modification peaks in early mitotic extracts and declines in later stage extracts [37]. That temporal pattern parallels that observed here for Aurora B-SUMO2/3 PLA products (Figure 2A) and is consistent with the localisation of RANBP2 during mitosis: when the nuclear envelope dissolves at mitotic entry, RANBP2 localises to the spindle microtubules, a fraction is gradually delivered to kinetochores as microtubules to establish contact with them. Thus, RANBP2 reaches its highest concentration at kinetochores in metaphase, when chromosomes are fully aligned [26,27,28] and PIAS 3 detaches from them [39]. In early anaphase, RANBP2 remains at kinetochores and detaches from them in late anaphase/telophase to be recruited around the segregating chromosomes [26], and our unpublished data. Therefore, a significant RANBP2 fraction is in close proximity to both Aurora B and Borealin at kinetochores in the prophase-to-metaphase window, during which both proteins form SUMOylated products. We actually found that RANBP2 forms PLA ligation products with Aurora B, mostly localised between the spindle microtubules and CREST-stained kinetochores in prometaphase and metaphase (a representative prometaphase is shown in Figure 5A), suggesting that the two proteins can interact therein.

PIAS3 was previously identified as the SUMO E3 ligase responsible for Aurora B SUMOylation [39]; PIAS3 silencing abolished Aurora B SUMOylated bands detected in reactions with co-transfected, overexpressed components (i.e., FLAG-tagged Aurora B and His6-tagged SUMO-2), yet only a minor population of the overall cellular pool of Aurora B molecules appeared to be SUMO-conjugated in these assays. That quantitative observation, together with the timing of PIAS3 detachment and RANBP2 enrichment at kinetochores in metaphase, when Aurora B-SUMO 2/3 products reach their highest abundance, raised the possibility that RANBP2 might also be implicated in the SUMOylated state of Aurora B. To address that question, we silenced RANBP2 in synchronised cultures by double thymidine block and release, as described (Figure S4A), to obtain cultures highly enriched in mitotic stages (see Figure S4D). As a read out of RANBP2 silencing, both SUMO-RANGAP1 (Figure S4B,C) and SUMO-borealin (Figure 5B) showed dramatically reduced levels, as expected. Under these same conditions, Aurora B-SUMO2/3 PLA signals were also significantly down-regulated in prometaphase and metaphase (Figure 5C). We conclude therefore that, in addition to PIAS3, RANBP2 is also implicated in the SUMOylation state of Aurora B. 

We reasoned that RANBP2 silencing, which down-regulates Aurora B/SUMO 2/3 products, should reproduce at least some of the phenotypes associated with the Aurora B^K202R^ mutation. Indeed, we found that RANBP2 silencing impaired the concentration of endogenous Aurora B at centromeres in prometaphase and metaphase, and signals overlapped in part with the DAPI-stained chromosomal profile (Figure S5A, quantified in Figure S5B). RANBP2 silencing instead did not affect the localisation of Aurora B at the midbody in telophase. 

**Figure 6:**
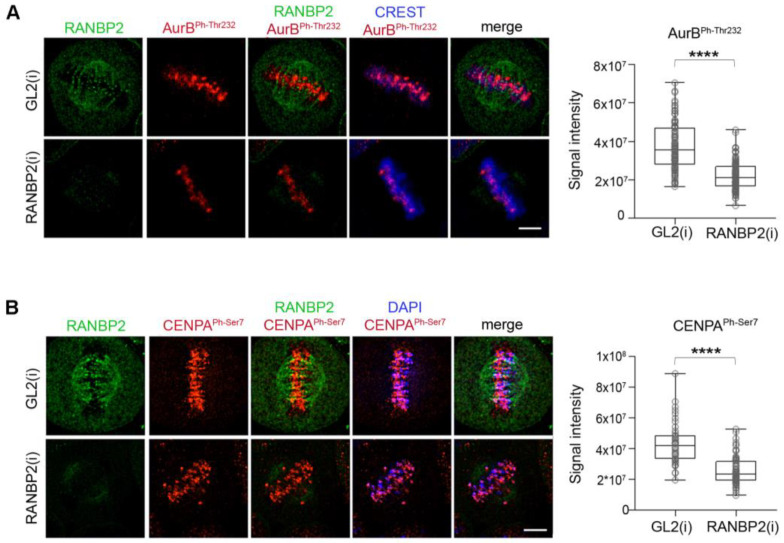
RANBP2 silencing impairs Aurora B autophosphorylation at Thr232 (**A**) and phosphorylation of CENP-A at Ser7 (**B**) at centromeres. Experiments were repeated three times. The quantitative analysis of markers in control (interfered with neutral GL2 siRNAs) and RANBP2-interfered cultures is shown in the box plot and the statistical analysis is as follows: AurB^phThr232^ (n, 100 for GL2 and 107 for RANBP2), **** *p* < 0.0001; CENP-A^ph-ser7^ (n, 45 and 72 cells) **** *p* < 0.0001.

As previously seen for SUMO-null Aurora B^K202R^, the reduced Aurora B concentration at centromeres in RANBP2-silenced metaphases did not reflect a failure to interact with CPC members, as INCENP/Aurora B PLA reactions similarly occurred in the presence or absence of RANBP2 (Figure S5C). We also found that both centromere-associated Aurora B^Ph-Thr232^ (Figure 6A) and CENP-A^Ph-Ser7^ signals (Figure 6B) significantly decreased in RANBP2-interfered metaphases compared to controls. Thus, RANBP2 silencing generates phenotypes recapitulating those observed with SUMO-null Aurora B^K202R^ mutant, strengthening the conclusion that RANBP2 contributes to SUMO conjugation, localisation and activity of Aurora B at prometaphase/metaphase centromeres.

## 4. Discussion 

In this work, the development of in situ intramolecular PLA has enabled us to build a precise spatial and temporal map of PLA products formed by endogenous Aurora B and SUMO2/3 peptides during mitosis in intact human cells. In cells examined at various stages of mitotic progression, PLA products accumulated at centromeres in prometaphase and metaphase, then were substantially down-regulated. Thus, the temporal span of SUMOylation is restricted compared to that of Aurora B phosphorylation, and, in particular, does not involve midbody-associated Aurora B, indicating that SUMOylation distinguishes a specific window of Aurora B function at centromeres. 

SUMO2 and PIAS 3 ligase were previously identified as key elements in Aurora B SUMOylation, as PIAS3 SUMOylates Aurora B in reactions with overexpressed components, and PIAS3 silencing abolished SUMOylated Aurora B forms. However, it has been reported that purified Aurora B, when incubated in vitro with SUMO conjugation components, cannot be detected in the SUMOylated state [39], suggesting that either post-translational modifications (PTMs), and/or stabilising partner(s) may be required for Aurora B SUMOylation in eukaryotic cells. Among PTMs, the most obvious would be phosphorylation, which facilitates SUMOylation in other proteins and marks Aurora B activity. We have found, however, that Aurora B activity, and hence phosphorylation, is not required for SUMOylation, as Aurora B-SUMO products effectively form in the presence of the specific inhibitor ZM447439 (our unpublished data, J.B., M.D. and P.L. not shown). Furthermore, in eukaryotic cells only a small subpopulation of Aurora B is found to be SUMO-conjugated at steady state, strengthening the idea that a stabilising factor may contribute to Aurora B SUMOylation; those observations led the authors to hypothesise that a SIM-containing factor specifically interacts with SUMO-conjugated Aurora B [39]. The identification of RANBP2 in our study would fulfil that expectation. Here we find that inactivating RANBP2 drastically reduced Aurora B-SUMO2/3 PLA products, indicating that RANBP2 also contributes to the formation of stable Aurora B-SUMO products at centromeres until metaphase. These circumstantial lines of evidence support the idea that RANBP2 adds a level of control to the stability and/or local dynamics of SUMOylated Aurora B species at centromeres/kinetochores.

Both SUMO2 and PIAS 3 show midbody-localised fractions in cytokinesis [39]. Endogenous Aurora-B/SUMO PLA products, however, are hardly detectable at that stage, indicating that the mere proximity of Aurora B to these components is not sufficient for Aurora B/SUMO stable PLA product formation. The down-regulation of SUMO-Aurora B PLA products in anaphase coincides temporally with RANBP2 detachment from kinetochores and relocation around the segregating chromosomes [26,28,31]. The decrease in SUMOylated Aurora B would therefore be consistent with SENP2 activity becoming predominant over RANBP2 at that point.

RANBP2 recruitment from nuclear pores to microtubule-attached kinetochores during mitosis depends on nuclear transport receptors [28] and on the nuclear pore subcomplex NUP107-160 [54]. The NUP107-160 subcomplex itself, comprising nine nucleoporins, translocates as an entire unit at kinetochores at NE breakdown [55,56]. SENP2, the SUMO-deconjugating enzyme for Aurora B, is also a shuttling protein associated with nuclear pores during most of the cell cycle [57,58] and targeted to kinetochores in mitosis, to which it is tethered via NUP107-160. Interestingly, SENP2 overexpression impairs chromosome congression in a manner that specifically depends on its kinetochore targeting [9]. Thus, NUP107-160 orchestrates the localisation of both SENP2 and RANBP2, which have antagonistic functions in the SUMO pathway, i.e., deconjugation and direct SUMO ligation or stabilisation, respectively. It is possible that the recruitment of SUMOylation factors with opposed activities is related to the turn-over of SUMOylation on target proteins at kinetochores and centromeres as attachments take place. Interestingly, earlier work also implicated the NUP107-160 subcomplex in the accumulation of CPC members at kinetochores and showed that Aurora B localisation at kinetochores was impaired in cells lacking the crucial Seh1 component of the NUP107-160 subcomplex [59]. In retrospect, the capacity of NUP107-160 to localise Aurora B may be mediated, at least in part, by SUMO-Aurora B stabilisation by kinetochore-associated RANBP2. 

In previous studies, the SUMO acceptor site, lysine K207 in murine Aurora B [38] and K202 in human Aurora B [39], was mutagenised and, despite its highly conserved position of the kinase, different effects were reported. In the present work, the mutation of lysine K202 impaired the concentration of human Aurora B at centromeres in prometaphase and metaphase, causing its retention at chromosomes arms together with fractions of INCENP and borealin; it also rendered Aurora B catalytically defective in in vitro assays. Chromosomal retention of SUMO-null Aurora B, similar to the pattern described here, was reported for the murine mutant Aurora B^K207R^, which was reported to be kinase-proficient [38]; that result was taken as an indication that SUMOylation primarily governs Aurora B localisation. In *C. elegans*, SUMOylated Aurora B homologue, AIR-2, was also detected by PLA in metaphase, and inactivation of individual SENPs suggested that SUMOylation is necessary for AIR-2 extraction from chromosomes [40]. These lines of evidence suggest a role of SUMOylation in facilitating Aurora B mobilisation from chromosomes in early mitosis. In previous experiments, a catalytically inactive Aurora B mutant, when expressed at high levels, also impairs centromere localisation and mislocalises its partners at chromosomes [47], indicating that levels of expression of Aurora B mutants are critical to their ability to assume their correct localisation. Human Aurora B^K202R^ mutant proved catalytically defective [39 and this paper] and could not be used to separate the consequence of loss of SUMOylation from those caused by the loss of kinase activity. We sought to circumvent this issue by examining the endogenous Aurora B under various conditions. Up-regulating Aurora B activity using a Kif11 inhibitor showed that this was paralleled by a corresponding increase in SUMOylated Aurora B, indicating that the two modifications can progress in parallel during mitosis. Conversely, RANBP2 silencing, which reduced endogenous Aurora B SUMOylation to almost unmeasurable levels, concomitantly induced an increased retention of Aurora B and CPC partners at chromosomes and impaired its concentration at centromeres; this was accompanied by reduced autophosphorylated Aurora B and phosphorylated CENP-A at centromeres. These observations are consistent with the idea that SUMOylation facilitates Aurora B detachment from chromosomes, and that the interaction with RANBP2 at centromeres stabilises the SUMO-modified form therein, facilitating its full activation. Interestingly, induction of SENP2 overexpression as a tool to increase Aurora B SUMO deconjugation, hence going in the same direction as RANBP2 silencing, also yielded reduced autophosphorylated Aurora B^phThr232^ [39]. It is worth noting that while the K202R mutation abolishes Aurora B activity as it may distort Aurora B structure near the activation loop, neither RANBP2 silencing nor SENP2 overexpression directly affect Aurora B structure, but both impair its SUMO-conjugated state. 

In summary, the results reported here suggest that SUMOylation is linked to centromere-associated Aurora B, while ruling out a role of SUMO in cytokinesis. They also show that RANBP2 regulates and/or stabilises Aurora B-SUMO2/3 products at centromeres in prometaphase and metaphase. Together with the previously established role of RANBP2 for Borealin SUMOylation, the data might hint at a role of RANBP2 as an additional regulator providing fine tuning to the control of CPC dynamics at centromeres. 

## Data Availability

All data are available from the authors upon request.

## Supplementary Materials

The following supporting information can be downloaded at: https://www.mdpi.com/article/10.3390/cells12030372/s1, Table S1: Antibodies used in this work; Figure S1: Specificity controls for intramolecular PLA assays between Aurora B and SUMO2/3; Figure S2: Generation of stable cell lines expressing RNAi-resistant Aurora B^WT-GFP^ and Aurora B^K202R-GFP^ in a dox-inducible manner; Figure S3: AuroraB^K202R^ mutant interacts with CPC partners; Figure S4: RANBP2 silencing in HeLa cells; Figure S5: RANBP2 silencing impairs Aurora B concentration at centromeres.
