# Supplementary material for: Aurora B SUMOylation Is Restricted to Centromeres in Early Mitosis and Requires RANBP2
Source: Cells. 2023 Jan 19;12(3):372. doi: 10.3390/cells12030372 (PMC9913629; doi:10.3390/cells12030372)
Supplement: Supplementary file 1 [file cells-12-00372-s001.zip › cells-2130576-supplementary.pdf]

Aurora B SUMOylation is Restricted to Centromeres in Early Mitosis and Requires RANBP2

Erica Di Cesare 1,2,†,‡, Sara Moroni 1,2,†,§, Jessica Bartoli 1,2,†,‖, Michela Damizia 1,2,¶, Maria Giubettini 3, Carolin Koerner 4, Veronica Krenn 4,, Andrea Musacchio 4 and Patrizia Lavia 1,2,***

1. *Institute of Molecular Biology and Pathology (IBPM), CNR National Research Council of Italy, 00185 Rome, Italy*
2. *Department of Biology and Biotechnology "Charles Darwin", Sapienza University of Rome,*
3. *00185 Rome, Italy*
4. *CrestOptics, 00165 Rome, Italy*
5. *Max Planck Institute of Molecular Physiology, 44227 Dortmund, Germany*

* Correspondence: patrizia.lavia@cnr.it, patrizia.lavia@uniroma1.it

† These authors contributed equally to this work.

‡ Present address: Stony Brook School of Medicine, Stony Brook, NY 11794, USA.

§ Present address: Special Product's Line SpA, 03012 Anagni, Italy.

‖ Present address: Istituto di Ricerca in Biologia Molecolare, IRBM Science Park, 00071 Pomezia, Italy.

¶ Present address: Department of Cellular, Computational and Integrative Biology (CIBIO), University of Trento, 38123 Trento, Italy.

** Present address: Department of Biotechnology and Biosciences, University of Milan-Bicocca, 20126 Milan, Italy.

Running title: Aurora B SUMOylation in early mitosis

Keywords: SUMOylation, Aurora B, in situ proximity ligation assay (isPLA), RANBP2, mitosis

SUPPLEMENTARY TABLE S1. ANTIBODIES USED IN THIS WORK

Immunofluorescence and PLA

PRIMARY	HOST	SOURCE
alpha-tubulin	mouse	T5178, Sigma-Aldrich
alpha-tubulin	rat	ab6161, Abcam
alpha-tubulin	chicken	ab89984, Abcam
Aurora B	rabbit	ab2254, Abcam
Aurora B	mouse	611082, Becton Dickinson
Aurora BphThr232	rabbit	636102, BioLegend
Aurora BphThr232	mouse	600-401-677S, Rockland
Borealin	rabbit	ab74473, Abcam
Borealin	mouse	ab67126, Abcam
CREST	human	15-234-0001 Antibodies Inc
CENP F	rabbit	NB 500-101, Novus Biologicals
CENPA	mouse	ab13939, Abcam
CENP A phThr3	rabbit	NL41, Millipore
gamma-tubulin	mouse	T6557, Sigma-Aldrich
H3 phSer10	mouse	3H10, Millipore
INCENP	mouse	ab23956, Abcam
INCENP	mouse	B-4, SC-376514, Santa Cruz Biotechnology Inc.
RANBP2	mouse	D-4 sc-74518, Santa Cruz Biotechnology Inc.
SUMO2/3	mouse	M114-3, MBL Life Science
TPX2	rabbiy	NB 500-179, Novus Biologicals

SECONDARY	CONJUGATION	SOURCE
anti-mouse	Texas Red	Vector
anti-mouse IgG	AlexaFluor 647	Jackson Imm.
anti-mouse IgG	AMCA	Jackson Imm.
anti-mouse IgG	Rhodamine	Jackson Imm
anti-rabbit IgG	CY3	Jackson Imm.
anti-rabbit IgG	FITC	Santa Cruz
anti-chicken IgG	Alexa 647	Jackson Imm.
anti-human IgG di	AMCA	Jackson Imm.
anti-mouse/anti-rabbit	CY 3.5	www.olink.com/products/duolink/how-use-duolink
anti-mouse/anti-rabbit	CY3	www.olink.com/products/duolink/how-use-duolink

Western immunoblotting

PRIMARY	HOST	SOURCE
actin	goat	I-19 sc1862, Santa Cruz Biotechnology Inc.
alpha-tubulin	mouse	B512, Sigma- Aldrich
Aurora B	rabbit	N-ter 1-100, ab2254 Abcam
GFP	rabbit	ab6556, Abcam
GFP	mouse	11814460001, Roche
NUP153	mouse	QE5 AB81463, Abcam
RANBP2	rabit	N-20, sc-15442 Santa Cruz Biotechnology Inc.
RANGAP1	mouse	N-19, sc-1862 Santa Cruz Biotechnology Inc

SECONDARY	CONJUGATION	SOURCE
anti-rabbit IgG	HRP	sc-2004, Santa Cruz Biotechnology Inc
anti-mouse IgG	HRP	sc-2005, Santa Cruz Biotechnology Inc
anti-goat IgG	HRP	sc-2354, Santa Cruz Biotechnology Inc

SUPPLEMENTARY FIGURE LEGENDS

Figure S1. Specificity controls for intramolecular PLA assays between Aurora B and SUMO2/3 peptides. A. Positive controls for PLA reactions of Aurora B with established interacting partners, Borealin and INCENP. **B.** As a negative control, PLA amplification yielded no signal for the Aurora B and gamma-tubulin pair. **C.** RNA interference-mediated Borealin silencing in HeLa cells. After quantitative IF, the extent of Borealin depletion, relative to control cultures interfered with neutral siRNA (GL2), was quantified in the box plots (**** $p<0.0001$, extremely significant difference; Mann-Whitney test, 60 counted cells / group, 2 experiments).

Figure S2. Generation of stable cell lines expressing RNAi-resistant Aurora B^{WT}-GFP and Aurora B^{K202R}-GFP in a dox-inducible manner. **A.** Map of wild-type and K202R mutant Aurora B: both are rendered RNAi-resistant by introducing three close synonymous substitutions in the coding sequence (double arrowhead line) and can therefore be expressed in an Aurora B-silenced background. The coding sequences also carry in-frame Myc and GFP tags cloned downstream of a dox-inducible promoter. **B.** Western blot analysis (40 μg of protein extract per lane) of cells expressing either Aurora B^{WT}-GFP or mutant Aurora B^{K202R}-GFP RNAi-resistant sequences (78 KDa) probed with GFP antibody. – and + indicate the addition of Aurora-B specific siRNAs. A twin filter was probed with Aurora B antibody to control silencing of the endogenous Aurora B and selective expression of the constructs after dox induction in cells treated with Aurora B-specific siRNAs. Alpha-tubulin was used as a loading control. Only the regions where proteins of interest migrate are shown. **C.** Still images from video recorded cultures induced to express either wild-type or K202R Aurora B, monitored by the GFP signal appearance. **D.** Release from STLC-dependent inhibition of the kinesin kif11 activates Aurora B. IF to $AurB^{phThr232}$ shows that Aurora B activity is stimulated by STLC treatment (2 hours) and even further after STLC release (30 min) cells. The box-and-whiskers plot represent the distribution of $AurB^{phThr232}$ signals in individual cells from control (CTR) and STLC-released cultures (n, 80 cells, 2 experiments). Aurora $B^{phThr232}$ increased significantly in STLC release compared to CTR (**, $p<0.01$).

Figure S3. $AuroraB^{K202R}$ mutant interacts with CPC partners. **A.** INCENP PLA products were assessed in cells silenced for the endogenous Aurora B and expressing Aurora B^{K202R}: both Aurora B forms retain the ability to interact with INCENP. **B.** INCENP concentrates at centromeres in Aurora B^{WT}-GFP-expressing metaphase cells, while being largely diffuse over DAPI-stained chromosomes in metaphase cells expressing $AuroraB^{K202R}$-GFP. In anaphase and telophase both the Aurora B^{WT}/INCENP and the $AuroraB^{K202R}$/INCENP pairs localise at the midbody in an undistinguishable manner. **C.** Borealin signals concentrate at metaphase centromeres in cells interfered for the endogenous Aurora B and expressing Aurora B^{WT}-GFP; in contrast, cells reconstituted with Aurora B^{K202R}-GFP show impaired Borealin localisation at centromeres. All assays in the dox-inducible cell lines were repeated in 3 or 4 independent experiments.

Figure S4. RANBP2 silencing in HeLa cells. **A.** Protocol for RANBP2 silencing in HeLa cultures synchronised by thymidine arrest and release. Control cultures were treated with neutral siRNA, indicated as GL2(i). **B.** Western immunoblotting of protein extract (40 μg per lane) shows that RANBP2-specific siRNAs depleted the cells of RANBP2 as well as SUMO-conjugated RANGAP1 (signals are quantified in **C**); the nucleoporin NUP153 used for control dos not vary. Only the regions where proteins of interest migrate are shown. **D.** The panels show the enrichment in mitotic cells in synchronised cultures. Mitotic stages are identified from both their chromosome configuration (DAPI staining) and the spindle organisation (TPX2 staining). RANBP2 was homogeneously depleted in the interfered culture. 40x objective. **E.** High-resolution images showing a control metaphase, treated with neutral GL2 siRNA, with RANBP2 (green) at the spindle (TPX2, red) and at microtubule-attached kinetochores. Chromosomes are stained with DAPI. Below, TPX2 is unaffected in the RANBP2-interfered metaphase. 100 x objective.

Figure S5. RANBP2 silencing impairs Aurora B concentration at centromeres. **A.** In control cells interfered with neural siRNAs [GL2(i)], Aurora B IF signals (red) are centromere-restricted; in RANBP2-interfered cells, a chromosome-associated Aurora B fraction is evident in prometaphase and metaphase cells: the mask profile of DAPI (chromosomes) and Aurora B IF shows an almost total overlap in RANBP2-interfered panels. **B.** The histograms represent the percentage of cells displaying either centromere-restricted (CEN), or centromere/chromosome (CEN/CHROM) diffuse localisation of Aurora B in control and RANBP2-interfered samples. Differences are highly significant in prometaphase and metaphase cells (****, $p<0,0001$). Aurora B localisation at the midbody in anaphase and telophase does not vary, with or without RANBP2 (ns, non significant difference). At least 80 cells/samples were counted in 3 experiments and the frequency of patterns were compared using the $\chi 2$ test (two-sided). **C**. RANBP2 interference does not prevent the association of Aurora B with INCENP in PLA reactions.

A. Positive PLA controls

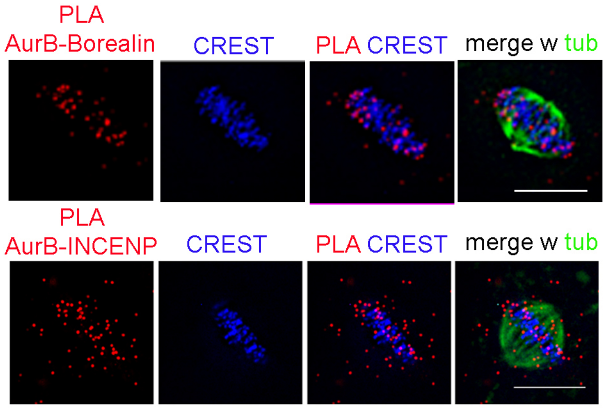

B. Negative PLA control

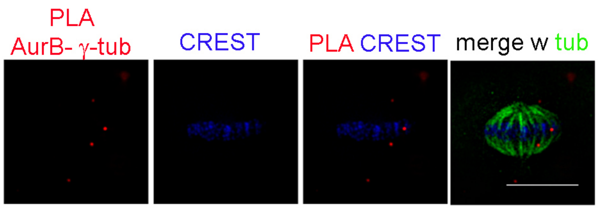

C. Borealin interference control

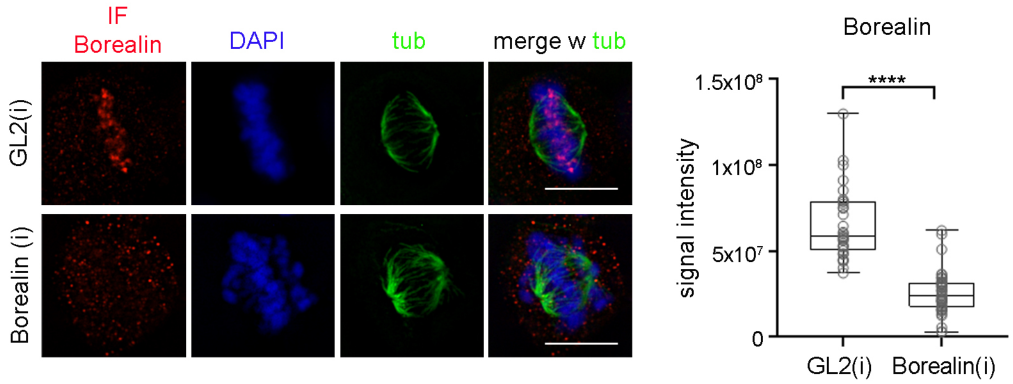

Figure S1

Figure S2

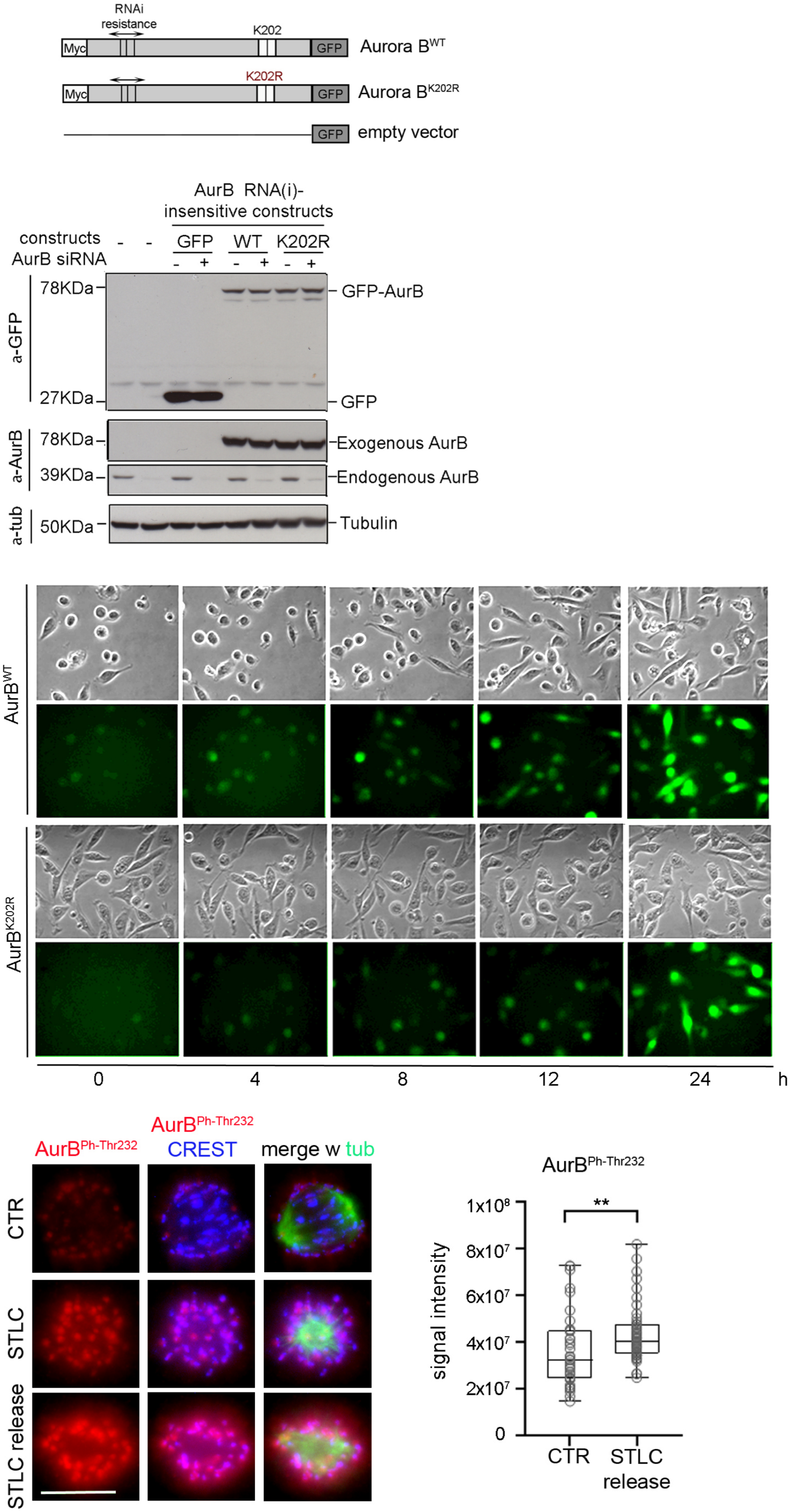

RNAi
resistance
K202
Myc
GFP
Aurora B^WT
K202R
Myc
GFP
Aurora B^K202R
GFP
empty vector
AurB RNA(i)-
insensitive constructs
constructs
AurB siRNA
GFP
WT
K202R
a-GFP
78KDa
27KDa
GFP-AurB
GFP
a-AurB
78KDa
39KDa
Exogenous AurB
Endogenous AurB
a-tub
50KDa
Tubulin
AurB^WT
AurB^K202R
0
4
8
12
24
h
AurB^Ph-Thr232
AurB^Ph-Thr232
CREST
merge w tub
CTR
STLC
STLC release
AurB^Ph-Thr232
signal intensity
1x10^8
8x10^7
6x10^7
4x10^7
2x10^7
0
**
CTR
STLC
release

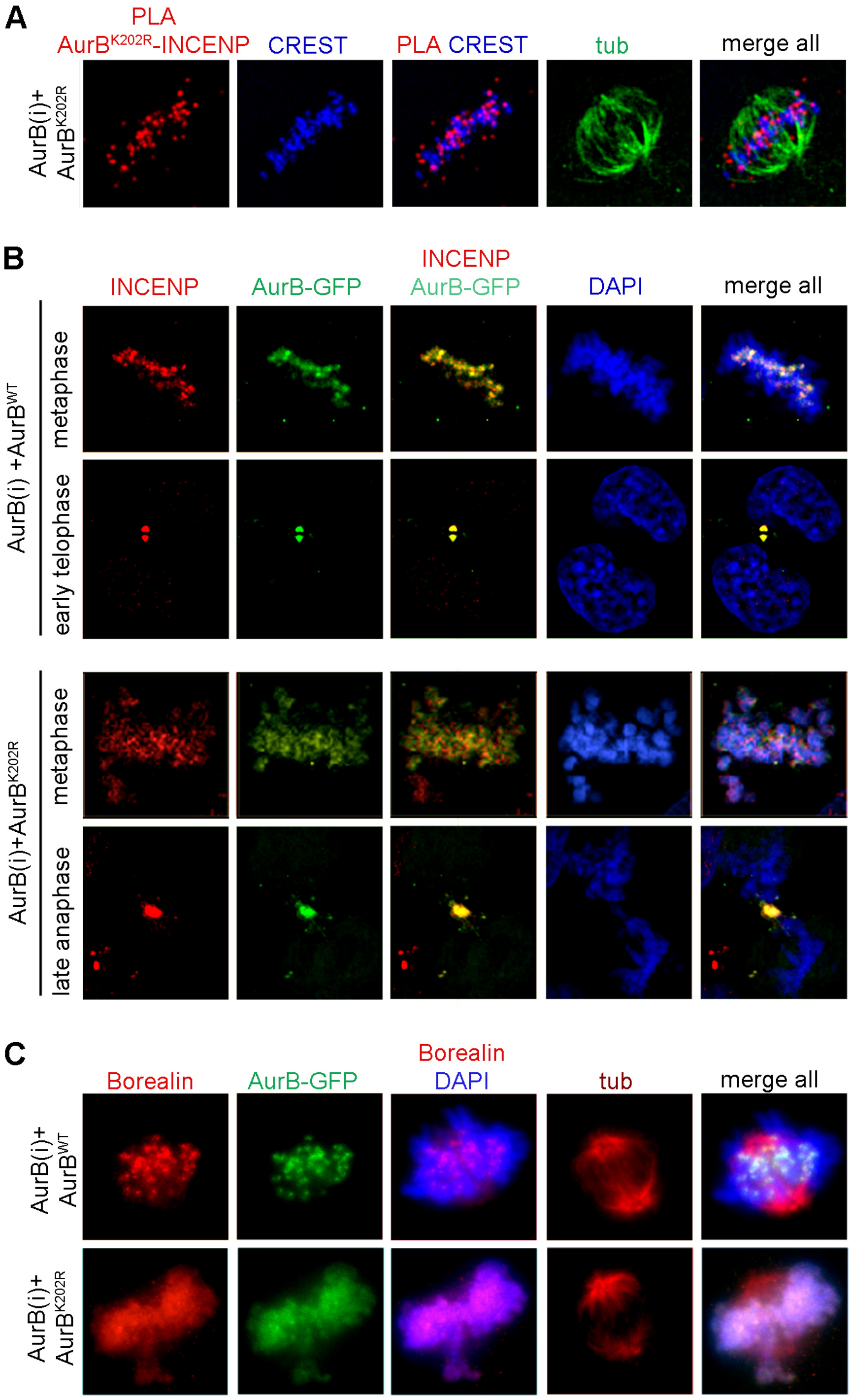
A
PLA
AurBK202R-INCENP
CREST
PLA CREST
tub
merge all
AurB(i)+
AurBK202R
B
INCENP
AurB-GFP
INCENP
AurB-GFP
DAPI
merge all
AurB(i) +AurBWT
metaphase
early telophase
AurB(i)+AurBK202R
metaphase
late anaphase
C
Borealin
AurB-GFP
Borealin
DAPI
tub
merge all
AurB(i)+
AurBWT
AurB(i)+
AurBK202R

Figure S3

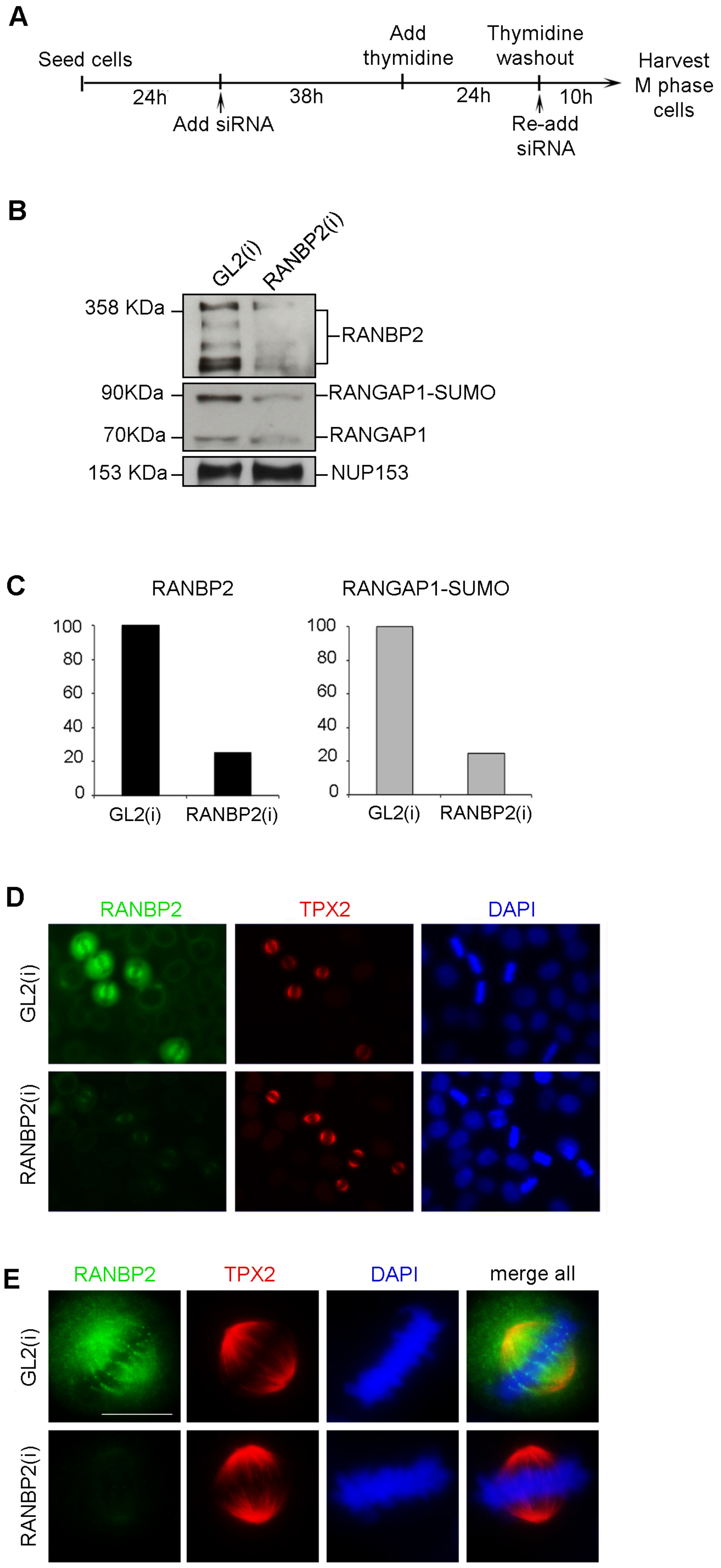
A
Seed cells
24h
Add siRNA
38h
Add
thymidine
24h
Thymidine
washout
10h
Re-add
siRNA
Harvest
M phase
cells
B
GL2(i)
RANBP2(i)
358 KDa
RANBP2
90KDa
RANGAP1-SUMO
70KDa
RANGAP1
153 KDa
NUP153
C
RANBP2
RANGAP1-SUMO
100
80
60
40
20
0
GL2(i)
RANBP2(i)
D
RANBP2
TPX2
DAPI
GL2(i)
RANBP2(i)
E
RANBP2
TPX2
DAPI
merge all
GL2(i)
RANBP2(i)

Figure S4

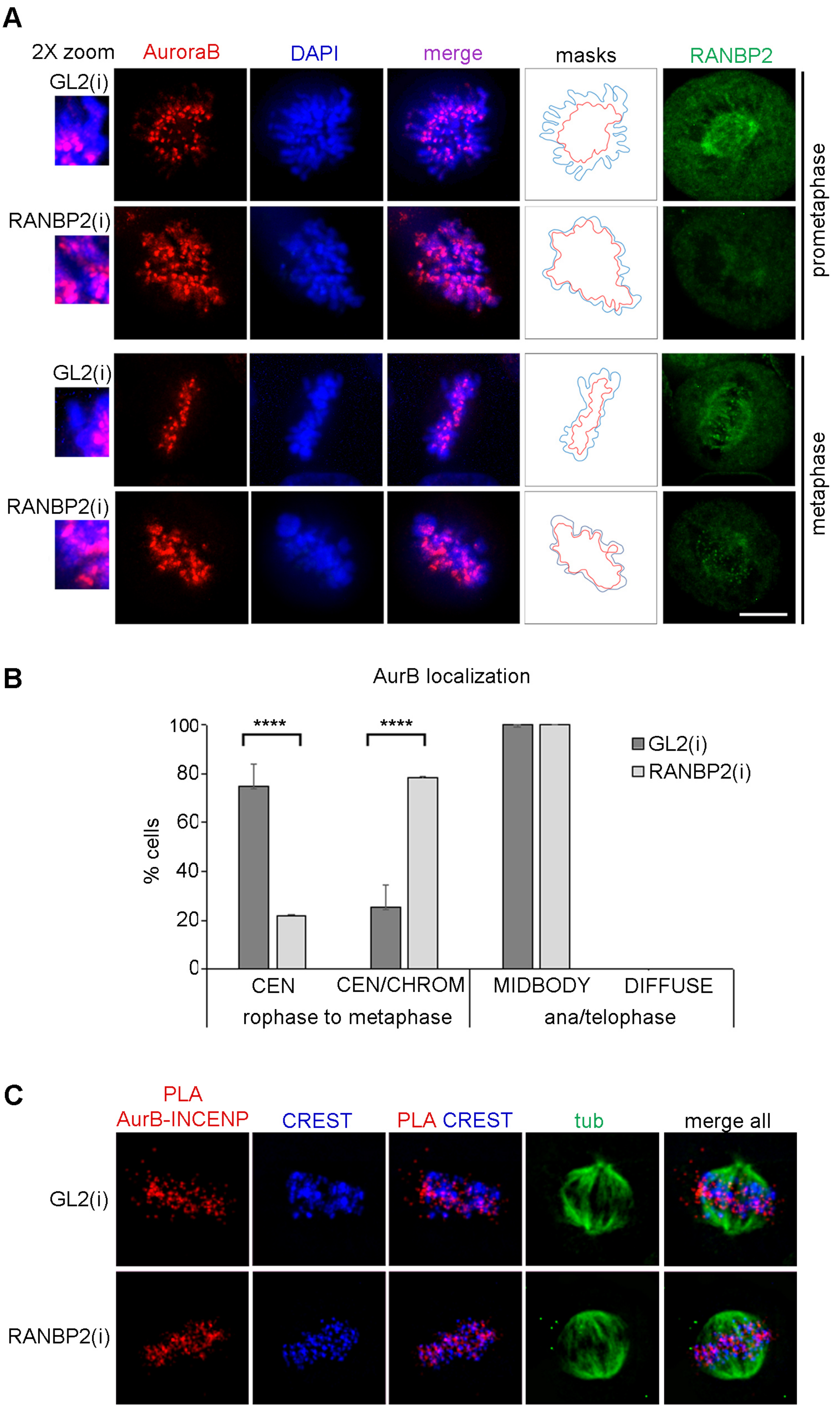

A
2X zoom
AuroraB
DAPI
merge
masks
RANBP2
GL2(i)
RANBP2(i)
GL2(i)
RANBP2(i)
prometaphase
metaphase
B
AurB localization
100
80
60
40
20
0
% cells
****
****
GL2(i)
RANBP2(i)
CEN
CEN/CHROM
MIDBODY
DIFFUSE
rophase to metaphase
ana/telophase
C
PLA
AurB-INCENP
CREST
PLA CREST
tub
merge all
GL2(i)
RANBP2(i)

Figure S5
